# Incremental Growth Analysis of a Cantilever Beam under Cyclic Thermal and Axial Loads

**DOI:** 10.3390/ma17184550

**Published:** 2024-09-16

**Authors:** Ali Shahrjerdi, Hamidreza Heydari, Mehdi Bayat, Mohammadmehdi Shahzamanian

**Affiliations:** 1 Mechanical Engineering Department, Malayer University, Malayer 84621-65741, Iran; hamidreza.est.1374@gmail.com; 2 Cvili Engineering Department, Aalborg University, 9220 Aalborg, Denmark; bayat_me@yahoo.com; 3 Department of Mechanical Engineering, McMaster University, Hamilton, ON L8S 4L7, Canada; mmshahzamanian@gmail.com

**Keywords:** ratcheting, cyclic thermomechanical load, finite element, beam, thermal loading

## Abstract

Ratcheting analysis for cantilever beams subjected to the thermomechanical loads is presented using the finite element method. The cantilever beam is constrained along the vertical direction, and plane stress conditions are assumed according to the bilinear isotropic hardening model. Two points are considered to obtain areas of ratcheting by using linear extrapolation. The results and output diagrams for ratcheting with elastic-perfect plastic behavior are illustrated. It was revealed that the beam behaves elastically after the first considerable plastic strain, which is seen in two shakedown regimes. The numerical results are verified with known and analytical results in the literature. The results indicate a strong correlation between the outcomes from the cyclic ANSYS Parametric Design Language (APDL) model and Bree’s analytical predictions. This consistency between the finite element analysis and the analytical solutions underscores the potential of finite element analysis as a powerful tool for addressing complex engineering challenges, offering a reliable and robust alternative to traditional analytical methods.

## 1. Introduction

Nowadays, the ratcheting behavior of cantilever beams under cyclic thermal and axial loads has become a critical phenomenon in engineering structures such as pipelines and pressure vessels in chemical plants, where accurate modeling is essential for ensuring structural integrity and safety. Many practical engineering segments in force and chemical plants are exposed to extreme loading conditions due to the cyclic thermal and mechanical loads they must be designed to endure. If cyclic and constant thermomechanical loads induce stress in the plastic domain, the components could lead to failure due to the accumulation of excessive deformation, known as ratcheting or fatigue, as mentioned in [1,2,3,4]. Some researchers conducted an experimental test and found convincing methods to show the ratcheting frontier appropriately. In 2006, Bingjun Gao [5] pointed out that the ratcheting frontier determined by employing the C-TDF approach, with the altered Jiang–Sehitoglu model, truly isolates the shakedown area. In 2012, Shariati et al. investigated the ratcheting and fatigue of polyacetal under uniaxial cyclic loading [6]. The test information showed that the ratcheting strain and strain rate ratcheting is sensitive to the applied stress amplitude and the mean stress, affecting the polyacetal’s fatigue life. In 2017, Zhu et al. studied mean stress and ratcheting corrections in the fatigue life prediction of metals [7]. They proposed a model delivering the highest and desired accuracy and sturdy life predictions with the lowest model prediction errors.

Much attention has been devoted to cylinder components, which are widely used in power and chemical plants, and very often they are subjected to internal pressure and repeated thermal stress that is mentioned in [8,9] and other related papers [10,11,12]. In 2017, Shariati and Kolasangiani conducted experimental tests with a servo-hydraulic Instron 8802 machine available on the laboratory of Ferdowsi University of Mashhad in Iran and studied the ratcheting behavior of SS304L cylindrical shells under cyclic combined and uniaxial loadings [13]. They showed that as the angle of the cylindrical shell increases, the accumulation of plastic deformation becomes more extensive because of the increase in the bending moment. The combination of cyclic thermal loads with different types of mechanical loads, torsion, and tension is highly investigated to identify the accumulation strain of cylinders by using mathematical and numerical methods, which are performed by Bree [14] and Hyeong-Yeon Lee and [15]. Different structures and geometries have different results in ratcheting phenomena. Some authors worked on parallel bar structures [16,17], a simplified complex structure model. In 1964, Parkes discussed the problem of thermal ratcheting in an aircraft wing due to cyclic thermal stresses superimposed on the normal wing loads [18]. The wing is simulated by an elastic-perfect plastic two-bar structure in which the temperature of one of the bars (representing the skin) is cycled between two limits. In contrast, the temperature of the other bar (representing the web) is kept constant at the lower temperature limit. In addition to cylindrical components and bar assembly, quadrangular components such as beams [19,20,21] and plates [22,23,24] are the most common members of the industry. S. I. Shahraini et al. [25] discussed the generalized differential quadrature (GDQ) and dynamic relaxation (DR) methods to develop the generalized differential dynamic relaxation (GDDR) method for analyzing elastic–plastic plates. Validated through theoretical analysis, Abaqus simulation, and experiments, GDDR reduced nodes, computational time, and iterations by about 80% and improved stress calculation accuracy with a 70% reduction in the mean squared error, using only three nodes per direction compared to the eight needed by other methods. M. Servatan et al. [26] aimed to evaluate the ratcheting response of additively manufactured (AM) samples of Al 4043, SS316L, and Ti–6Al–4V alloys using hardening rules and finite element analysis. Utilizing Ahmadzadeh-Varvani (A-V) kinematic hardening and Lee-Zavrel (L-Z) isotropic hardening, yield surface evolution was assessed, with Chaboche’s model applied for finite element analysis. The predicted and simulated ratcheting curves showed close agreement but fell below the measured values, indicating a comprehensive evaluation of hardening models but some discrepancy between simulation and actual measurements. B. Yu et al. [27] aimed to investigate the fatigue properties of Ti6Al4V alloy rhomboidal dodecahedral cell structures produced by electron beam melting for medical bone implants. In addition, they homogenized the structures with different mapping ratios and examined their compressive fatigue behavior and failure mechanisms under high cyclic loading, including post-treatment by homogenization to reduce residual stress. The authors showed in their results that the mapping ratio significantly affected the mechanical performance and fatigue properties, with the fatigue life model providing insights into predicting fatigue behavior; strengths included the comprehensive analysis and modeling, while one weakness was the complexity of translating these findings to practical applications. G. Shyamala et al. [28] attempted to enhance beam-to-column connections in precast concrete frames using nano-silica. Also, the authors tested reinforced concrete joints with various combinations of fly ash and nano-silica, finding that 2.5% nano-silica improved compressive and flexural strength, addressing the strength reduction from fly ash. The researchers also demonstrated that nano-silica increased strength effectively, though a higher axial compression improved the bearing capacity but reduced the flexibility. H. Li et al. [29] aimed to investigate the flexural behaviors of NSC and HSC beams under monotonic and cyclic loadings. The authors performed three-point bending tests, proposed prediction formulas based on normalized EIR-CMODR curves, and improved the hysteresis curve models. Based on the results, the researchers showed accurate P-CMOD curve predictions, though further refinement of the XFEM adjustments was needed.

The results in this paper are verified based on Bree’s outputs [30]. Purely elastic regimes, shakedown regimes, and ratcheting regimes are the main phenomena that should be observed. In fact, there are several articles regarding shakedown [31,32,33]. In 2017, Spagnoli et al. used a non-linear programming set of rules primarily based on the static theorem of restriction evaluation to calculate the shakedown limit load [34]. They studied the shakedown of general two-dimensional discrete systems, including friction and plasticity. In 2018, Hartwig Hübel and Bastian Vollrath used the simplified theory of plastic zones as a direct approach, which is applied to simplifying a pipe band and a straight pipe, both subjected to combinations of several loads [35]. They proved that the concept is appropriate for delivering practical estimates of the strains accumulated in the state of elastic shakedown at the cost of some linear elastic analyses. As a matter of fact, all the materials could be considered temperature-dependent, and some researchers have investigated temperature-dependent ratcheting [36,37] and also temperature-dependent shakedown [9,38,39], although, in this paper, all the materials are temperature-independent. In addition, there is a vast range of material behaviors to describe ratcheting. Chaboche is one of the most common models that authors use in their studies [40,41,42]. Some authors have utilized Ohno–Wang kinematic hardening [43,44]. In 2010, Mohammad Abdel-Karim evaluated the performance of countless non-linear kinematic hardening rules and proved that none of the examined kinematic hardening guidelines is ordinarily sufficient to simulate all ratcheting responses for the experiments under consideration [45].

Research studies indicate that scientists use extensive methods that are relevant to study ratcheting and investigate the parameters with them. Some experimental research has been carried out to introduce new results [46,47]. In 2000, Sylvain Callocha et al. used experimental methods to study the mechanical behavior of 316 austenitic stainless steel under multiaxial loadings and to investigate ratcheting under tension–torsion nonproportional loadings [48]. In addition, some analytical methods have been utilized to obtain new results [16,49,50]. In 2011, R. Adibi-Asl and W. Reinhardt generalized the static shakedown theorem to allow the analysis of plastic shakedown. All of their results have been obtained analytically [51]. In 2016, M. Zeinoddini et al. dealt with the cyclic plastic response and strain ratcheting of circular steel tubes under repeated inelastic pure bending. They have evaluated analytical and experimental outcomes and determined reasonable agreement [52]. In 2017, Xiaotao Zheng et al. deduced the analytical ratchet limits of cylindrical pipelines subjected to numerous typical cyclic nonproportional loading combinations theoretically in keeping with the noncyclic technique [53]. Some authors have used the FEM method, even in the medical field, such as [54], to assess their results [55,56,57]. Radim Halama et al. described a new cyclic plasticity model based on the Abdel-Karim–Ohno kinematic hardening rule, Jiang–Sehitoglu memory surface, and modified Calloch isotropic hardening rule. The model was implemented in the commercial FE code ANSYS using a Fortran subroutine [58]. 

Overall, study in the field of ratcheting has been very popular in recent years [59,60,61]. In 2023, the impact of the thickness, cutout numbers, and various cutout shapes on the ratcheting behavior of 304 steel sheets exposed to cyclic axial loading was examined by Ali Shahrjerdi and Ali SafariFard, and essential results were given [11]. The authors found a good agreement among the numerical and empirical findings. In another research study [62], the impact of shear deformation was examined for thin-walled metal parts. The authors designed a device for cyclic shear experiments and employed a DP600 sheet to investigate the fatigue phenomenon under a shear path without buckling. In a major advance in 2023, Ali Shahrjerdi et al. investigated the issue of ratcheting in a functionally graded beam consisting of two types of steels and obtained a Bree diagram for better analysis [12]. Despite such interest, many gaps and shortcomings need to be tackled. Also, many authors have used APDL in their articles [63,64,65]. In 2016, Quoc Huy Vu and Dinh Quy Vu dealt with integrating fatigue criteria in finite element code using the APDL scripting language [66]. In 2018, Radim Halama et al. proposed a brand new technique of fatigue check assessment based totally on digital image correlation method [67]. Their simulation was conducted using APDL to verify the experimental results. As is evident, APDL is one of the most popular softwares that scientists utilize. A more detailed look at the other papers revealed that to the best of the authors’ knowledge, no work has been reported to date using APDL to perform ratcheting analysis and reveal a different area of material behavior due to thermomechanical load.

Previous works on ratcheting analysis often relied on simplified analytical models or limited numerical approaches that did not fully capture the complexities of cyclic thermal and axial loads. These methods frequently assumed idealized boundary conditions and material behaviors, which could not accurately predict the incremental growth of plastic strains and the resulting ratcheting phenomena in real-world scenarios. This study addresses these limitations by employing a comprehensive finite element approach with a bilinear isotropic hardening model, providing a more accurate and nuanced analysis of beam behavior under cyclic loading conditions. For instance, Wei et al. [68] examined welded stainless steel beam-to-column connections under cyclic loading and found that while hysteretic performance was good, energy dissipation decreased with larger sections. Numerical models effectively validated the experimental results. Chen et al. [69] developed a finite element model for CFRP-strengthened beams and found that increasing the sectional area and prestress improved the load capacity, with optimal prestress levels between 10% and 30%. CFRP plate profiles affected the failure modes but not the ultimate load. Wang et al. [70] tested high-strength RC beams and found that increased reinforcement improved the capacity, with minimal impact from concrete strength. They validated and adjusted code formulas for yield moments and crack widths and developed a new formula for predicting the ultimate moments. Le et al. [71] investigated CFST beams under monotonic and cyclic loads, finding that lower diameter-to-thickness ratios improved load capacities and stiffness, though they increased strength degradation. They proposed a simplified model for quickly estimating the moment capacity of CFST beams. Mussa et al. [72] found that CFRP strengthening, especially with strips and wraps, significantly improved RC beams’ load capacity and deflection resistance. The numerical models in LS-DYNA accurately predicted beam performance with minimal error. Merzouki et al. [73] examined thermal vibrations in functionally graded nanobeams and found that the thickness ratio, beam length, and thermal effects crucially affected the vibration behavior, highlighting the need to consider these factors in design. However, our work introduces a novel approach to ratcheting analysis by integrating advanced finite element modeling with cyclic thermal and axial loads, offering superior accuracy and insights compared to traditional analytical methods and previous studies. Also, a summary of the related works is given in Table 1.

In this paper, numerical analysis was conducted using the FEM and APDL codes to obtain precise results. The study focuses on the ratcheting behavior of a thin beam subjected to cyclic thermal loading and constant tensile stress. The elastic–plastic response of the beam under thermomechanical loading conditions was considered, with FEM employed to model the behavior. The APDL code was specifically developed to implement an iterative approach for ratcheting analysis, enabling the calculation of ratcheting and non-ratcheting boundaries. The primary objective of this research is to provide a comprehensive examination of ratcheting in cantilever beams under combined thermomechanical loads using FEM. The study utilizes bilinear isotropic hardening and elasto-perfect plastic models to predict the accumulation of plastic strain during cyclic loading and identify regions prone to ratcheting. The practical implications of this research are significant, particularly in engineering fields where structural components are frequently subjected to repeated thermal and mechanical stresses. Industries such as aerospace, civil engineering, and automotive manufacturing benefit from a deeper understanding of ratcheting behavior, as this knowledge can enhance the design and longevity of critical components, including bridges, aircraft wings, and vehicle frames. By accurately predicting ratcheting, engineers can design more robust and reliable structures, reducing maintenance costs and improving safety.

The findings of this study are presented across all domains of the interaction loading diagram, encompassing elastic, shakedown, fully plastic, and ratcheting regions. This research is distinctive in its application of FEM to analyze the ratcheting behavior of a cantilever beam under cyclic thermal and axial stresses using a bilinear isotropic hardening model. This approach integrates cyclic thermomechanical loading with comprehensive FEM analysis, providing detailed insights into ratcheting processes that are difficult to capture with conventional analytical methods. Additionally, the research introduces a generic, dimensionless framework by employing high theoretical values for material properties, making it applicable to various materials and conditions. This versatility underscores the potential of FEM in predicting the performance of advanced and emerging materials, marking a significant advancement in structural analysis and design. The analysis reveals a strong correlation between the cyclic APDL model and Bree’s analytical results, highlighting the reliability of finite element analysis (FEA) in addressing complex engineering problems. This supports FEA as a robust alternative to traditional methods. For instance, chemical plants, pipelines, and pressure vessels often experience cyclic thermal and mechanical loads due to temperature fluctuations and internal pressures, making accurate ratcheting predictions crucial for their safe operation.

## 2. Methodology

In this section, the methodology is described comprehensively.

### 2.1. Solution for Stresses and Strains

**Figure 1:**
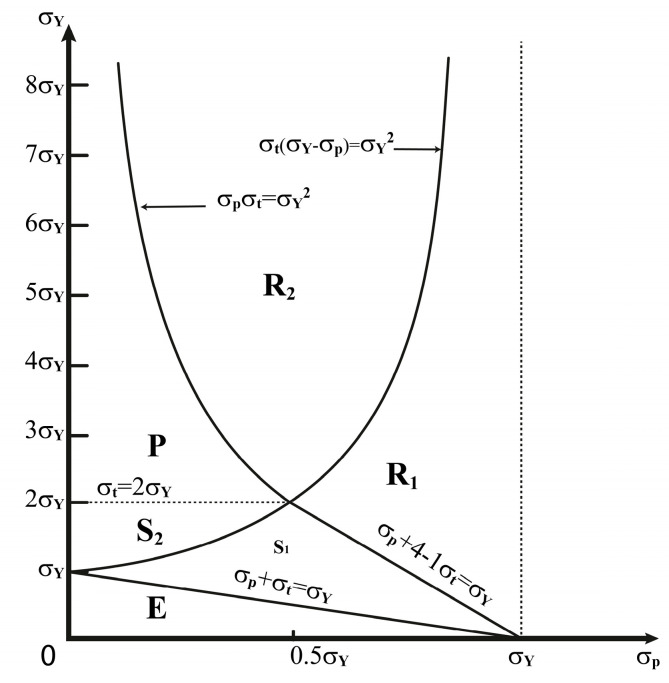
Load diagram for the uniaxial stress model.

The model is constructed by assuming plane stress conditions. Without a loss of generality, the temperature gradient can be chosen to vary between any reference’s values to the maximum value. The magnitude values of the temperatures and material properties are not important because of the dimensionless interaction diagram results. As is evident, the trend of stress–strain development is essential. Bree produced an analytical solution to the elastic–plastic behavior of thin tubes subjected to internal pressure and intermittent high-heat fluxes. He introduced six regimes related to the strain behavior of his structure, which can be seen in the load diagram in Figure 1. These regimes are as follows:

1. Purely elastic regime (E);
2. Shakedown regime one (S1);
3. Shakedown regime two (S2);
4. Plastic cyclic regime (P);
5. Ratcheting regime one (R1);
6. Ratcheting regime two (R2).

The structure’s behavior depends on where point $\left(\sigma_{y},\sigma_{t}\right)$ is located in the load diagram. Purely elastic behavior is experienced in stress regime E. Shakedown occurs after plastic increment deformation at the first half-cycle in the stress regimes S1 and S2. Plastic cycling happens in the stress regime P, while ratcheting occurs if $\left(\sigma_{y},\sigma_{t}\right)$ is a point in either of the stress regimes R1 or R2.

Bree ignored the axial stress in a thin tube, considered the stress in the hoop direction only, which is the dominant stress, and assumed a linear temperature distribution through the tube’s thickness. Finally, he reduced the thin tube to a slab, prevented bending, and subjected it to uniaxial stress and a cyclic thermal load. According to his assumptions to simplify the problem, Bree’s outputs can verify the results of the assumed beam in this study.

### 2.2. Material Behavior Assumptions

The elastic–plastic model with bilinear isotropic hardening was chosen for this study due to its computational efficiency and simplicity, which are two critical considerations for the initial investigation of ratcheting behavior in cantilever beams subjected to cyclic thermal and axial stresses. This model effectively depicts critical aspects of material behavior, such as the transition from elastic to plastic deformation and the subsequent hardening process. This simplified model reduces the complexity associated with more complex material behaviors, enabling a more focused investigation into the fundamental mechanisms of ratcheting and the identification of the shakedown regimes. This method enables a more comprehensive understanding of the fundamental response patterns and establishes a basis for future research incorporating more complex models.

It is recognized that most actual materials exhibit more intricate behaviors, including nonlinear hardening and varying degrees of anisotropy, even though the elastic–plastic model functions as an initial step. The bilinear hardening model is a frequently employed approximation in engineering applications for predicting material response under cyclic loading conditions, as it efficiently balances computational demands and accuracy despite its limitations. In order to more precisely depict the intricate behaviors of specific materials, future studies can incorporate more sophisticated material models such as those that account for rate-dependent effects and nonlinear hardening. Nevertheless, the elastic–plastic model was determined to be adequate for the objectives of this study in order to investigate the fundamental aspects of ratcheting and to verify the finite element methods against the existing literature. The bilinear isotropic hardening model is assumed. The yield criteria of this material behavior is as follows [30]:

(1) $$\left|\sigma \right|=\sigma_{y}\;(in\;plastic\;region)$$

(2) $$\left|\sigma \right|<\sigma_{y}\;(in\;elastic\;region)$$

where $\sigma_{y}$ is the yield stress. Thus, |σ| cannot exceed $\sigma_{y}$, and plastic strains are introduced to maintain the stress at its limiting value in regions where it exceeds the yield stress elastically.

The data presented in Table 2 are used for a dimensionless analysis, in which specific numerical values have been selected to simplify the study and facilitate comprehension without explicitly representing real-world data.

The theoretical nature of this investigation is underscored by the substantially large values, except for the thermal conductivity and Poisson’s ratio. This method enables the methodology to be applied to any material, irrespective of its specific mechanical properties, by allowing for the generalization of findings. The primary benefit of this method is its adaptability; it is capable of analyzing the ratcheting behavior of a diverse spectrum of materials with various characteristics, rendering it a valuable instrument for various engineering applications. Furthermore, all material properties in this investigation were presumed to be temperature-independent. Table 2 provides a comprehensive list of the specific material properties that were employed in the beam calculations.

The assumed yield strength of 30,000 MPa, along with the other material characteristics presented in Table 2, significantly exceeds the typical values observed in most engineering materials, which generally exhibit yield strengths in the range of several hundred megapascals. These assumptions are deliberately chosen to explore the theoretical limits and behavior of materials under extreme conditions, providing insights that could be relevant to the development of future advanced materials, whether emerging or yet to be discovered. For instance, the use of an exceptionally high yield strength allows for the investigation of potential behaviors and failure mechanisms in materials that might be produced using advanced composite techniques or nanotechnology.

These assumptions are inherently theoretical, offering assumptions to provide a baseline for understanding fundamental ratcheting mechanisms within a controlled and simplified framework. This simulation study establishes a foundation for subsequent investigations incorporating more realistic material properties. By initially employing these elevated material properties, the study ensures that complexities and unpredictable behaviors do not obscure the ability to clearly identify the elastic–plastic transition, hardening behavior, and ratcheting effects. This theoretical approach not only validates the finite element method and the fundamental concepts of ratcheting analysis but also prepares the groundwork for future research that will apply these methods to materials with properties more representative of actual engineering applications.

Our work aims to propose a novel conceptual model that, while grounded in current scientific principles, explores possibilities that extend beyond the limitations of existing materials and technologies. It is essential to recognize that scientific progress often hinges on exploring theoretical concepts that may take time to be verifiable but serve as a catalyst for future innovation and discovery. Including hypothetical materials in our study is a deliberate approach to stimulating discussion and exploration within the scientific community. While not currently realizable, these materials are based on theoretical principles that align with the fundamental laws of physics and materials science. Though not immediately verifiable, our model’s assumptions are constructed with rigorous attention to existing scientific knowledge and logical consistency. These assumptions are intended to provide a foundation for theoretical exploration, offering a framework that can be tested and refined as experimental techniques advance. Despite its limitations in experimental verification, the model presented offers a valuable contribution to the ongoing discourse in this field. Theoretical models often serve as a precursor to experimental breakthroughs, offering new insights and guiding the development of future technologies.

### 2.3. Problem Definition 

**Figure 2:**
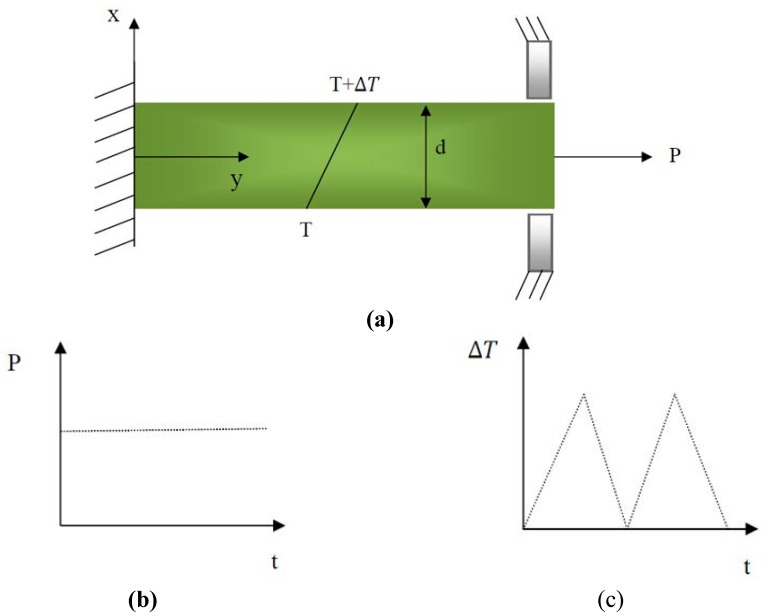
(**a**) Cantilever beam and applied loads. (**b**) Constant mechanical load. (**c**) Cyclic thermal gradient.

The cantilever beam is modeled in ANSYS 16 and constrained along the vertical direction to avoid deflection deformation. The beam is subjected to a uniaxial stress σ of mean value $\sigma_{p}$ resulting from a constant tensile load p and cyclic thermal stress resulting from a cyclic linear temperature gradient through the thickness, which changes linearly from T to T + Δ*T* from one side to another side. The deformation of the beam is considered just in the horizontal direction because of the existence of supports at the ends to prevent bending in the vertical direction when both applying and removing temperature gradients, which leads to thermal stress $\sigma_{t}$. The temperature at the upper side $x=\frac{d}{2}$ (if the co-ordinate x is assumed from the mid-thickness of the beam upwards) increases from T to T + Δ*T* during the first half of each cycle and then decreases from T + Δ*T* to T during the second half. The model and loads are shown schematically in Figure 2 below.

The cantilever beam was assumed to be in a plane tension state due to its resemblance to a thin-walled element. In this context, the use of analysis methods typically applied to sheet metal or membranes is justified by the fact that stresses perpendicular to the plane are deemed negligible compared to those within the plane. The coefficient of thermal expansion governs the dilatation (expansion or contraction) that temperature changes induce in the material. This is a notably significant aspect of this analysis. When a material is unrestricted in its ability to expand or contract, it experiences natural deformation due to temperature fluctuations, which leads to minimal or no thermal stresses. Nevertheless, the material experiences substantial thermal stresses when restrictions are imposed, such as fixed supports or attachments that limit unrestricted deformation. These stresses result from the material’s internal resistance to the constraints that impede its natural thermal expansion or contraction.

The cantilever beam depicted in Figure 2 is affixed in a manner that permits it to deform flexibly in response to temperature fluctuations without any substantial external constraints. As a result, the beam can expand or contract in response to temperature fluctuations, resulting in thermal stresses that are negligible or relatively low. In contrast, substantial thermal stresses would be generated if the beam were fixed or partially constrained, prohibiting free thermal deformation. The internal forces generated as the material resists the imposed constraints would result in these stresses, which could potentially cause structural damage or failure if not properly accounted for in the design. The analysis is simplified by the fact that the stresses in the thickness direction (Z direction) are negligible compared to those in the plane (X and Y directions) under plane stress conditions. The primary uniaxial stress is applied in the X direction, as illustrated in Figure 2. However, the resultant deformation and constraints in the vertical direction (Y direction) induce stresses in both the X and Y directions. This is under the plane stress assumption, which assumes that only in-plane stresses are taken into account and that those perpendicular to the plane are negligible. Therefore, the plane stress assumption and the uniaxial loading condition are compatible within the context of this analysis.

**Figure 3:**
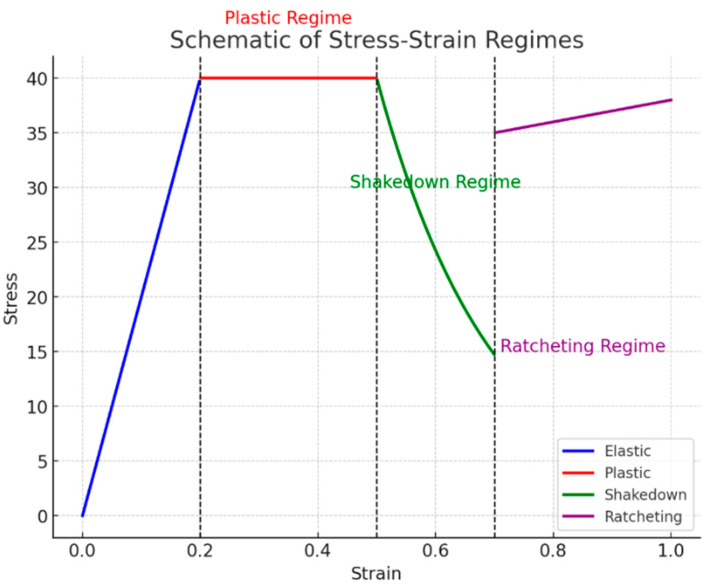
The schematic of stress–strain regimes.

Figure 3 illustrates the progression of material through different stress–strain regimes, starting with the elastic regime, where the material experiences a linear increase in stress proportional to strain, depicted by the blue line, indicating that the material returns to its original shape upon unloading.

As the strain reaches a threshold at around 0.2, the material enters the plastic regime, represented by a red line, where the stress remains constant despite increasing strain, signifying permanent deformation. Beyond this, at approximately 0.5 strain, the material transitions into the shakedown regime, shown by the green line, where the stress begins to decrease as the material undergoes adjustments to accommodate the applied loads, potentially leading to a stabilized condition without further plastic deformation. Finally, as the strain exceeds 0.7, the material enters the ratcheting regime, indicated by the purple line, where stress slightly increases again, reflecting a cumulative plastic deformation with each load cycle, which can eventually lead to material failure if the loading continues. The vertical dashed lines mark the boundaries between these regimes, providing a clear visualization of the points at which the material behavior shifts from one regime to another. This schematic effectively captures the complex behavior of materials under varying stress and strain conditions, offering insights into the different phases of material response in engineering applications.

### 2.4. The Study Procedure

The results are indicated by FEM and verified by theoretical results. Bilinear isotropic hardening was applied, and suitable structural and thermal mass elements were selected. The von Mises yield criterion could also be chosen to investigate problems concerning ratcheting. The criterion is isotropic and independent of hydrostatic pressure, which is the convincing first approximation criterion for materials like metals, polymers, and soaked geological materials.

The ANSYS Parametric Design Language (APDL) is used to program the whole simulation process. The von Mises yield criterion is as follows [30]:

(3) $$f\left(\sigma ,\sigma_{y}\right)=\sigma_{e}-\sigma_{y}=0$$

where $\sigma_{e}$ is the von Mises effective stress, and $\sigma_{y}$ is the yield strength, corresponding to the yield in uniaxial stress loading [30].

(4) $$\sigma_{e}=\sqrt{\frac{3}{2}\left(\sigma :\sigma -\frac{1}{3}tr{\left(\sigma \right)}^{2}\right)}$$

In this simulation, $\sigma_{t}$ can be obtained from Equation (5) [30]:

(5) $$\sigma_{t}=\frac{E\alpha ∆T}{2(1-\nu )}$$

where α denotes the coefficient of thermal expansion, quantifying the amount a material expands per unit length per degree of temperature increase. The maximum thermal stress can be used to characterize the temperature gradient as shown below [30]:

(6) $$\sigma_{t}=\frac{E\alpha ∆T}{2}$$

Equation (6) is used to obtain temperature gradients and simplify calculations. $\sigma_{p}$ and $\sigma_{t}$ must be selected according to Bree’s diagram in Figure 1.

**Figure 4:**
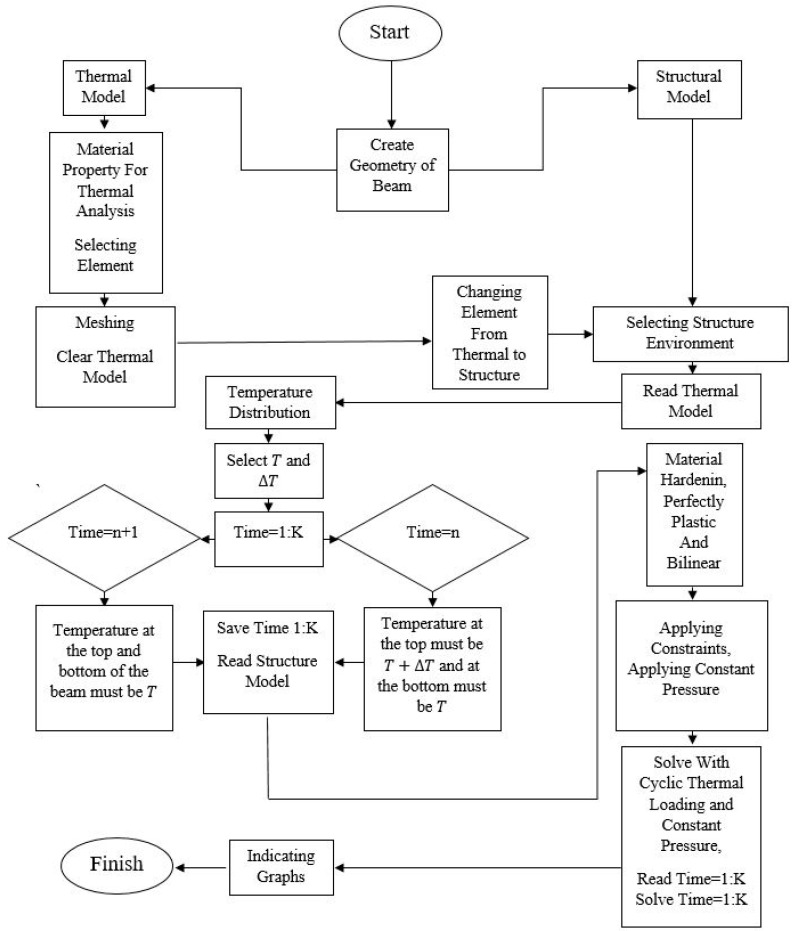
The steps in APDL.

To summarize the process of the FEM method, the flow chart below gives information about the stages that should be performed in order to obtain reasonable results. All the stages must be written using the ANSYS Parametric Design Language (APDL) as shown in Figure 4.

## 3. Results and Discussion

**Figure 5:**
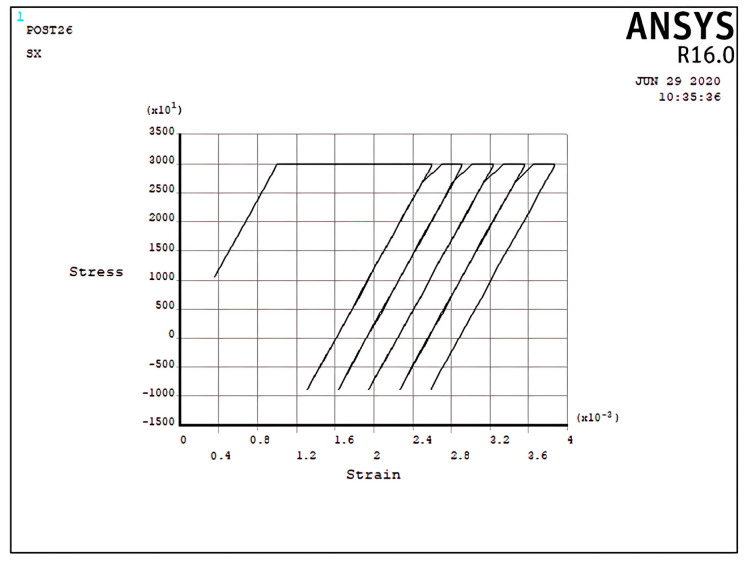
The stress–strain path corresponding to the stress regime R1 at $\frac{\sigma_{p}}{\sigma_{y}}=0.7,\;\frac{\sigma_{t}}{\sigma_{y}}=1.5$, $x=\frac{d}{2},$ and ∆T=3K.

**Figure 6:**
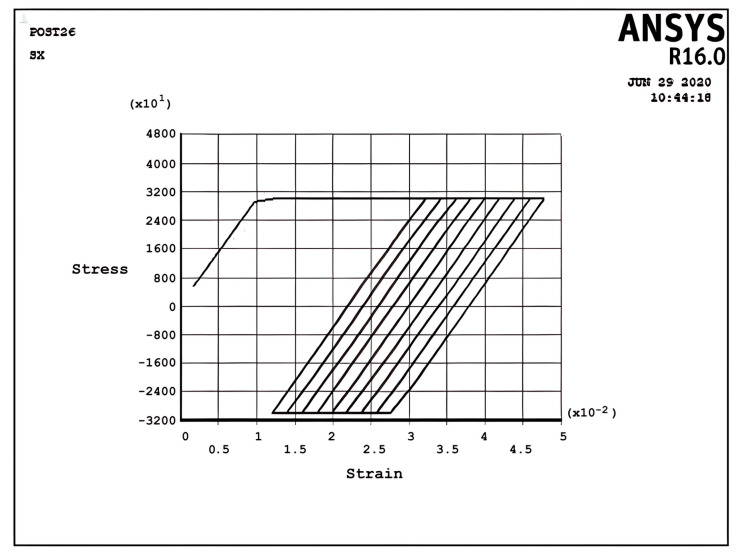
The stress–strain path corresponding to the stress regime R2 at $\frac{\sigma_{p}}{\sigma_{y}}=0.366,\;\frac{\sigma_{t}}{\sigma_{y}}=3$, $x=\frac{d}{2},$ and ∆T=6K.

**Figure 7:**
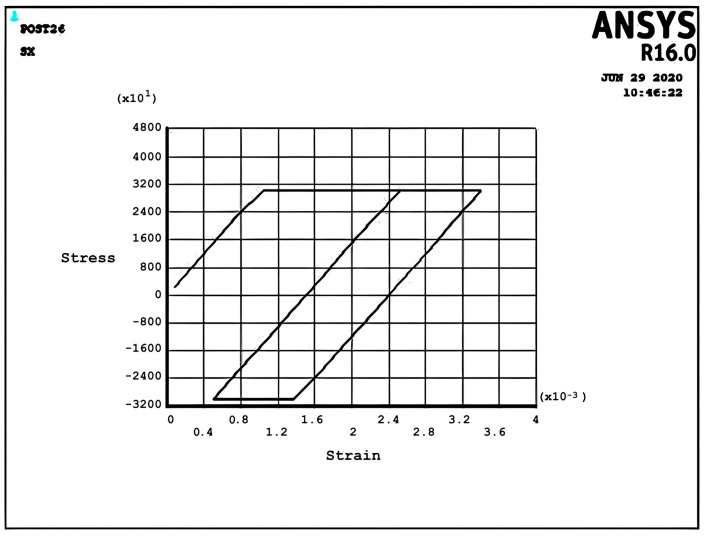
The stress–strain path corresponding to the stress regime P at $\frac{\sigma_{p}}{\sigma_{y}}=0.167,\;\frac{\sigma_{t}}{\sigma_{y}}=3$, $x=\frac{d}{2},$ and ∆T=6K.

**Figure 8:**
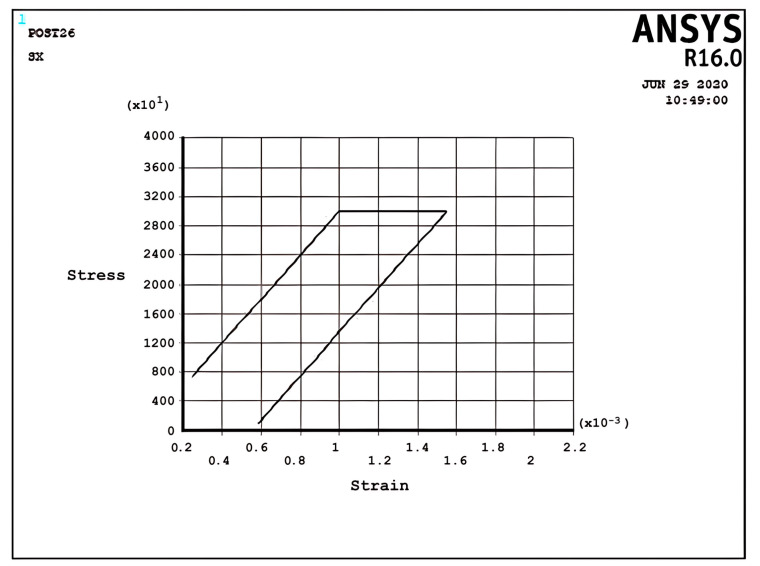
The stress–strain path corresponding to the stress regime S1 at $\frac{\sigma_{p}}{\sigma_{y}}=0.5,\;\frac{\sigma_{t}}{\sigma_{y}}=1$, $x=\frac{d}{2},$ and ∆T=2K.

**Figure 9:**
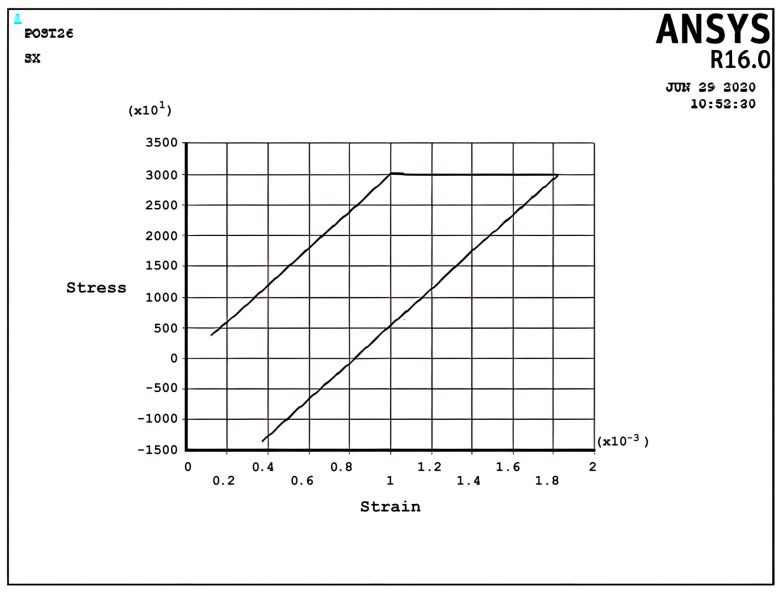
The stress–strain path corresponding to the stress regime S2 at $\frac{\sigma_{p}}{\sigma_{y}}=0.25,\;\frac{\sigma_{t}}{\sigma_{y}}=1.5$, $x=\frac{d}{2},$ and ∆T=3K.

**Figure 10:**
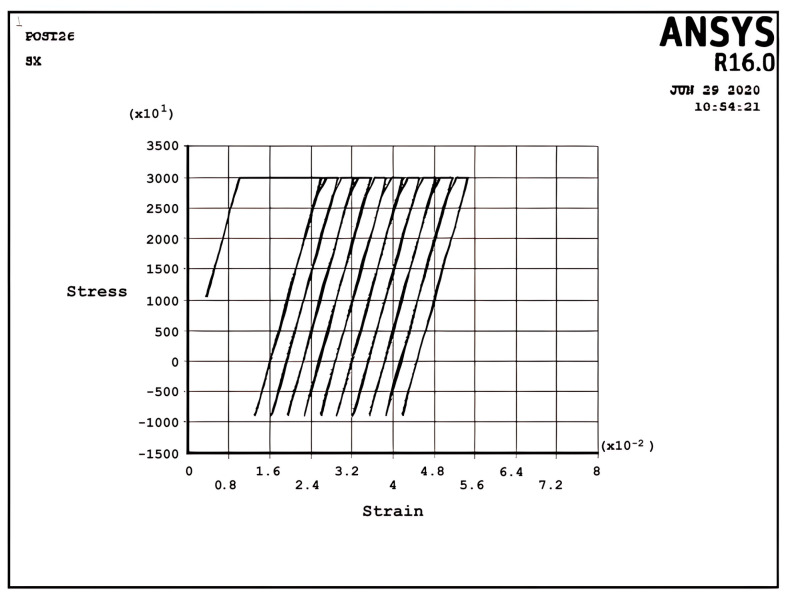
The stress–strain path corresponding to the stress regime R1 at $\frac{\sigma_{p}}{\sigma_{y}}=0.7,\;\frac{\sigma_{t}}{\sigma_{y}}=1.5$, $x=\frac{d}{2},$ and ∆T=3K with more cycles.

**Figure 11:**
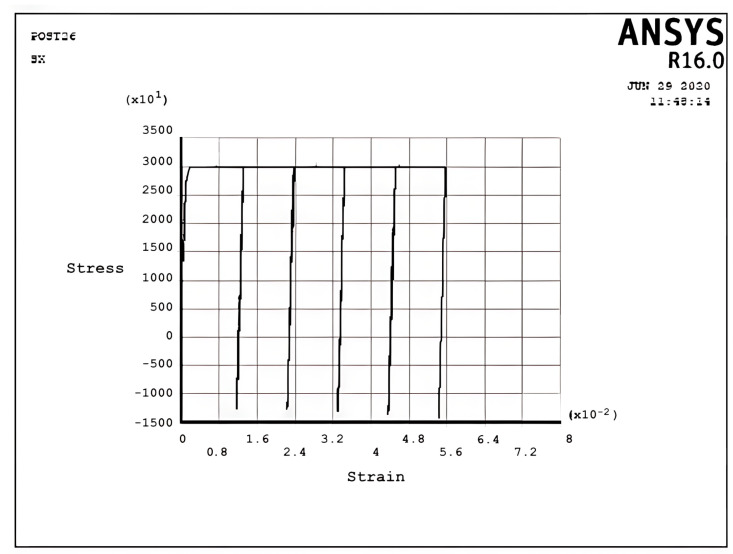
The stress–strain path corresponding to the stress regime R1 at $\frac{\sigma_{p}}{\sigma_{y}}=0.9,\;\frac{\sigma_{t}}{\sigma_{y}}=7$, $x=\frac{d}{2},$ and ∆T=14K.

The elastic–plastic results of the cantilever beam, which is prevented from bending in the five regimes (R1, R2, S1, S2, and P), are programmed by APDL and shown in Figure 5, Figure 6, Figure 7, Figure 8, Figure 9, Figure 10 and Figure 11. In these figures, the stress and strain relations of a node at the upper side of the beam $=\frac{d}{2}$ are calculated. The bilinear isotropic hardening model is invoked. The thermal cyclic load value for the analyses is obtained by Equation (6). Figure 5, Figure 6, Figure 7, Figure 8, Figure 9, Figure 10 and Figure 11 show the stress–strain diagrams extracted. Figure 5 shows the stress–strain path corresponding to the ratcheting regime R1, where the non-dimensional constant and non-dimensional thermal load $\frac{\sigma_{p}}{\sigma_{y}}=0.7,\frac{\sigma_{t}}{\sigma_{y}}=1.5$ for nodes at $=\frac{d}{2}$.

As can be seen, the start point begins from the non-zero stress value because of the constant load; consequently, the cyclic thermal load results in the accumulation of strain. At the first half-cycle, which means applying a thermal load, there is a large plastic strain in tensile yielding, and for the second half-cycle, which means unloading of thermal load, the beam shows only elastic behavior; therefore, there is plasticity only in the first half-cycles. However, the ratchet strains obtained in the first cycle are larger than those obtained in the subsequent cycles. Moreover, the ratchet strains for the second and subsequent cycles are identical. It is worth mentioning that the plastic deformation in the first half-cycle of the applied thermal load is equal to the total strain of the successive applied half-cycles. It can be noted that the difference in yield stress due to the tensile thermal load and initial tension stress because of the constant load equals the difference in the ultimate stress due to the compressive thermal load and initial tension stress because of the constant load. The interaction effect between the existence of the constant load and the bilinear isotropic hardening behavior of the model can explain this phenomenon. Figure 6 presents the stress–strain path corresponding to the ratcheting regime R2 at $\frac{\sigma_{p}}{\sigma_{y}}=0.366,\frac{\sigma_{t}}{\sigma_{y}}=3$, $x=\frac{d}{2}$.

In the ratcheting regime R2, Figure 6, the accumulation of strains can be identified. Similar to the ratcheting regime R1, the ratcheting strain in the first cycle is larger than that in the other cycles, which have the same ratchet strain. However, the differences between Figure 5 and Figure 6 belong to the yielding area. In Regime R1, Figure 5, only tensile yielding occurs; however, in Regime R2, both tensile and compressive yielding occur for the thermomechanical load; unlike the results of Figure 5, the algebra summation of maximum stress in tension and compression is zero. It is noted that the plastic strain when applying a thermal load is greater than the plastic strain when removing the thermal load, which leads to ratchet strain. 

Figure 7 describes the stress–strain curve in the cyclic plastic regime P at $\frac{\sigma_{p}}{\sigma_{y}}=0.167,\frac{\sigma_{t}}{\sigma_{y}}=3$, $x=\frac{d}{2}$. It can be seen that fully reverse cyclic plastic deformation occurs after the first half-cycle, which means the hysteresis loop shifts to the right-hand side. In contrast to Figure 6, the plastic strain when applying a cyclic thermal load equals the plastic strain during removing the cyclic thermal load, which means that there is no ratchet strain and it is fully reverse plastic. This situation introduces the low-cycle fatigue problem because of the existence of the plastic area.

Figure 8 and Figure 9 demonstrate the shakedown stress–strain path corresponding to the stress regimes S1 and S2 for $\frac{\sigma_{p}}{\sigma_{y}}=5,\;\frac{\sigma_{t}}{\sigma_{y}}=1$, $x=\frac{d}{2}$ and $\frac{\sigma_{p}}{\sigma_{y}}=25,\;\frac{\sigma_{t}}{\sigma_{y}}=1.5$, $x=\frac{d}{2}$, respectively. The beam can show elastic behavior after the first plastic strain depending on the circumstances, the amount of pressure, and the thermal loads. This phenomenon is known as a shakedown.

Figure 8 shows that the stress–strain curve after the first applied load follows a straight line instead of a loop shape in successive cyclic loads. The elastic behavior of the beam will present after a large plastic strain, with all tensile stress. Figure 9 presents the same behavior as Figure 8, but here, there are tensile and compressive stresses. Figure 8 and Figure 9 show that elastic behavior is seen, but a large plastic strain is very important in design. According to Bree’s diagram, all the figures illustrated are based on five cycles, which indicate ratchet strains and a non-ratcheting boundary. In addition to these results, the cycles can be increased to 10 and even more to achieve new results, as mentioned in the following example for the stress regime R1 with previous data.

Figure 10 presents the same behavior as Figure 5, but it has five cycles more than Figure 5 and similar results. New data like $\frac{\sigma_{p}}{\sigma_{y}}=0.9\frac{\sigma_{t}}{\sigma_{y}}=7$ with five cycles could also be replaced with previous data, as shown in Figure 5, to obtain new results, which are shown in Figure 11.

As a matter of fact, a number of data can be used. However, the results must be in good agreement with Bree’s diagram. The data may not be necessarily $\frac{\sigma_{p}}{\sigma_{y}}$ and $\frac{\sigma_{t}}{\sigma_{y}}$, actually $\sigma_{p}$ and T and Δ*T* could be selected at first, according to which the $\sigma_{y}$ and yield stress are calculated. The border of the ratcheting area is very important in predicting structural behavior in design and manufacturing. For calculating the ratcheting boundary, the steady-state plastic strain per cycle $\left({∆\varepsilon }_{p}/∆N\right)$ should tend toward zero [74]. Eight and sixteen different amounts of $\frac{\sigma_{t}}{\sigma_{y}}$ and $\frac{\sigma_{p}}{\sigma_{y}}$ are estimated in the ratcheting region to calculate the boundary. The steady-state results computations to define ratcheting and non-ratcheting boundaries are summarized in Table 3.

**Figure 12:**
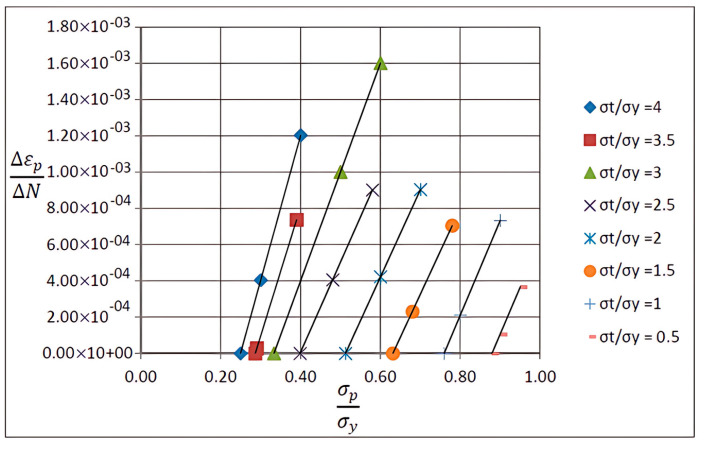
Variations in the ratchet strains with constant $\frac{\sigma_{t}}{\sigma_{y}}$ for the plane stress model.

The crossing points of the horizontal axis and oblique lines in Figure 12 demonstrate the maximum values of $\frac{\sigma_{p}}{\sigma_{y}}$, which cause no ratcheting. The linear extrapolation between the determined values of $\frac{\sigma_{p}}{\sigma_{y}}$ corresponds to a constant value of $\frac{\sigma_{t}}{\sigma_{y}}$. The linear extrapolation is acceptable because of the same slope of lines in Figure 12 by considering the generally small extrapolation amount [74].

**Figure 13:**
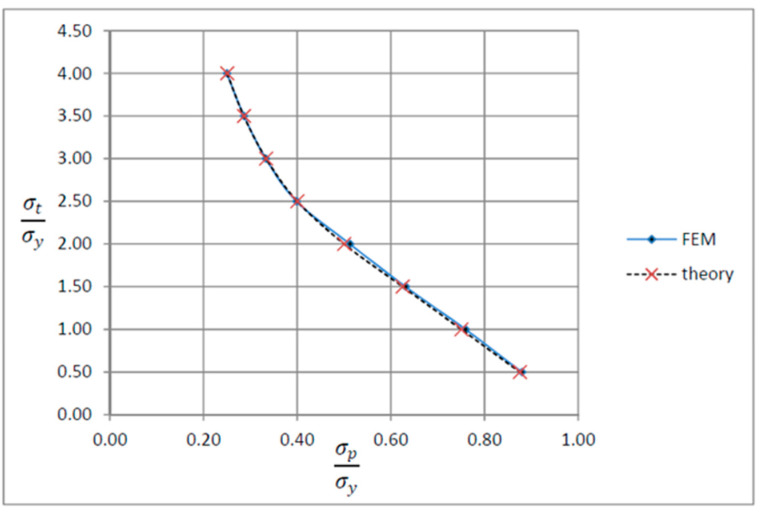
Bree interaction diagram, along with results from the theory.

Figure 13 shows the Bree interaction diagram obtained from the present results by FEM and an analytical solution from the following equation (Bree (1967) [30]):

(7) $$\sigma_{p}\sigma_{t}=\sigma_{y}^{2}\;\;\;\mathrm{if}\;\;\;\sigma_{t}\geq 2\sigma_{y}$$

(8) $$\sigma_{p}+\frac{1}{4}\sigma_{t}=\sigma_{y}\;\;\;\mathrm{if}\;\;\;\sigma_{t}\leq 2\sigma_{y}$$

As can be seen, a good agreement has been found between the results from the cyclic ANSYS Parametric Design Language (APDL) model and Bree’s results. Since there is an acceptable agreement between the finite element analysis results and the analytical results, we believe the finite element analysis will significantly aid researchers in solving any engineering problem. Overall, despite the fact that the analytical solution represents an innovative alternative to the other approaches, the finite element analysis has the potential to outperform all previous ones. The significant advantages of this analysis are as follows:

- Its remarkable capability to examine the critical design parameters prior to the construction process reduces potential costs.
- Its virtual prototyping capability allows the design process to be conducted with the fewest physical prototypes. Consequently, companies are less likely to spend such huge amounts of money on unsuccessful experiments.
- This analysis considers various conditions and characteristics (stress, fatigue, creep, ratcheting, shakedown, etc.) for the prototypes.
- By exploiting FEA, we will have high productivity levels in manufacturing, and the industries will considerably prosper.

Although the current analysis primarily concentrates on the ratcheting behavior of steel and its alloys under cyclic thermal and mechanical loading, it is crucial to acknowledge that comparable mechanisms are becoming more relevant to contemporary thermoplastic and composite polymer materials. The complex deformation behaviors that these materials exhibit, which are extensively used in aerospace, automotive, and energy applications, result from equivalent strain and thermal stresses. Therefore, further exploration is warranted. For example, Chakraborty et al. [75] reported that composite polymers exhibit substantial stress–strain responses during cyclic loading. These responses are influenced by factors such as the matrix properties and fiber orientation. Additionally, recent research conducted by Papadakis [76] and Arnaoutakis [77] has shown that a more comprehensive comprehension of the thermal–mechanical interaction in these advanced materials is required to address energy-efficient design considerations. The predictive accuracy of finite element models for thermoplastics and composites could be improved by incorporating such insights into the current framework, providing a more comprehensive understanding of material behavior across a broader range of engineering applications.

## 4. Conclusions

Incremental deformation analysis of a cantilever thin beam under constant mechanical load and cyclic thermal load is investigated using APDL coding. The bilinear isotropic hardening behavior material properties and plane stress conditions are considered. Constant horizontal mechanical load and cyclic linear temperature load through the thickness are coupled thermomechanical loads. Roller supports are applied at the right-hand side of the cantilever beam to omit any bending effects. APDL programming is used in ANSYS 16, and the iteration approach is used to predict the elastoplastic behavior of the beam uniaxially. The linear extrapolation method is applied to find the ratcheting and non-ratcheting boundary. All areas of the interaction loading diagram, such as shakedown, fully plastic, and ratcheting, are demonstrated. The results are compared with well-known results in the literature. Some salient observations of this research can be summarized as follows:

- For Regime R1, the plastic strain in the first applying thermal load equals the sum of the elastic and plastic strain of the applied load in the following cycles, as shown in Figure 5.
- As shown in Figure 5, the difference between the maximum and minimum stress related to a cyclic thermal load and the initial stress due to constant load in Regime R1 is equal.
- In contrast to the results of Regime R1, the absolute values of maximum and minimum stress are the same in Regime R2, as presented in Figure 6.
- Fully reversed plastic does not occur in Regime R2. When applying the thermal load, the plastic strain is larger than the plastic strain when removing the thermal load, whereas there is no plastic strain when removing the load in Regime R1.
- Low-cycle fatigue is found in Regime P, as shown in Figure 7.
- The elastic behavior after the first large plastic strain is seen in two regimes, S1 and S2, as shown in Figure 8 and Figure 9.
- All of the regimes with more cycles could also be observed, as shown in Figure 10.
- Figure 11 shows that various amounts could be obtained from Bree’s diagram to reach the regimes (elastic, shakedown, fully plastic, and ratcheting).
- Theoretical results were compared to results obtained by FEM, and it turned out that there is a good agreement between them, as shown in Figure 13.
- Based on the striking benefits of finite element analysis, exploiting this solution is recommended.

In fact, it was proved that the ANSYS Parametric Design Language (APDL) model could have the ability to illustrate ratcheting boundaries when data are not selected from Bree’s diagram. In addition, the results can be separated into five regimes, the same as Bree’s diagram, which is beneficial in the industry for predicting the behavior of a particular workpiece under different loadings. This phenomenon can be developed in the future. All material properties were temperature-independent in this paper, but they could be temperature-dependent to indicate new results, or the beam could be composed of different substances, each having its material properties. The findings of this finite element analysis are not experimentally confirmed as part of this research; nonetheless, they are evaluated against known analytical solutions, notably Bree’s results, to guarantee their correctness and dependability. While experimental verification is required to establish the results’ practical relevance, the primary goal of this work is to illustrate the suggested method’s theoretical viability and computational resilience. Future research will center on experimental validation, in which physical prototypes exposed to comparable cyclic thermomechanical stresses will be evaluated to compare with computational predictions. This ensuing experimental phase will aid in bridging the gap between theoretical analysis and real-world applications, confirming that the suggested FEM technique can accurately anticipate ratcheting behavior in realistic circumstances.

## Figures and Tables

**Table 1:** A summary of the related works.

Reference	Materials Studied	Experimental or Modeled	Main Tools Used	Key Results
Bingjun Gao [5]	Low carbon steel	Modeled	C-TDF approach, Jiang–Sehitoglu model	Isolated the shakedown area accurately.
Shariati et al. (2012) [6]	Polyacetal	Experimental	Uniaxial cyclic loading	Ratcheting strain sensitive to stress amplitude and mean stress.
Zhu et al. (2017) [7]	Metals	Modeled	Fatigue life prediction models	Proposed a highly accurate fatigue life prediction model.
Shariati and Kolasangiani (2017) [13]	SS304L cylindrical shells	Experimental	Servo-hydraulic Instron 8802 machine	Plastic deformation increases with cylindrical shell angle.
Bree (1967) [30]	Thin tubes	Modeled	Analytical methods	Introduced regimes related to strain behavior under cyclic loads.
Servatan et al. (2023) [26]	Al 4043, SS316L, Ti–6Al–4V alloys	Modeled	Finite element analysis (Chaboche’s model)	Close agreement between predicted and simulated ratcheting curves.
Yu et al. (2023) [27]	Ti6Al4V alloy (for medical implants)	Experimental and Modeled	Electron beam melting, fatigue life modeling	Mapping ratio significantly affects fatigue properties.

**Table 2:** Material properties of the beam.

Young’s Module	30 × 10^6^ MPa
Thermal conductivity	0.01 W/mK
Coefficient of thermal expansion	0.001/K
Poisson’s ratio	0.3
Yield stress	30,000 MPa

**Table 3:** The ratchet strains and ratcheting and non-ratcheting boundary for the model.

$\frac{\sigma_{t}}{\sigma_{y}}$	$\frac{\sigma_{p}}{\sigma_{y}}$	$\frac{{∆\varepsilon }_{p}}{∆N}$	$\mathrm{The}\;\mathrm{Boundary}\;(\mathrm{Calculated}\;\mathrm{through}\;\mathrm{Extrapolating})\;\frac{\sigma_{p}}{\sigma_{y}}$	$\frac{\sigma_{p}}{\sigma_{y}}$ (Theory)
4	0.3	0.04048 × 10^−2^	0.24936	0.25
	0.4	0.12044 × 10^−2^		
3.5	0.29	2.71 × 10^−5^	0.28618	0.28571
	0.39	0.07374 × 10^−2^		
3	0.5	0.10019 × 10^−2^	0.33298	0.3333
	0.6	0.16017 × 10^−2^		
2.5	0.48	0.04052 × 10^−2^	0.39841	0.4
	0.58	0.09018 × 10^−2^		
2	0.6	0.042242 × 10^−2^	0.51151	0.5
	0.7	0.09036 × 10^−2^		
1.5	0.68	0.02308 × 10^−2^	0.63155	0.625
	0.78	0.07042 × 10^−2^		
1	0.8	0.02119 × 10^−2^	0.75937	0.75
	0.9	0.7335 × 10^−2^		
0.5	0.9	0.10594 × 10^−3^	0.87969	0.875

## Data Availability

No new data were created or analyzed in this study.
